# Assessment of Auscultation Skills Among Junior and Senior Healthcare Providers for Pediatric Patients: A Retrospective Study

**DOI:** 10.7759/cureus.28495

**Published:** 2022-08-28

**Authors:** Rahaf Waggass, Bader Khawaji, Abdullah Abdulkareem, Abdulmjeed Alamri, Raed Ayoub, Mohamed Alaufey, Husain Alalgum, Ahmad Shamsuddin

**Affiliations:** 1 Cardiac Sciences, King Saud Bin Abdulaziz University for Health Sciences, Jeddah, SAU; 2 Basic Medical Sciences, King Saud Bin Abdulaziz University for Health Sciences College of Medicine, Jeddah, SAU; 3 College of Medicine, King Saud Bin Abdulaziz University for Health Sciences, Jeddah, SAU

**Keywords:** senior, junior, murmur, heart, echocardiogram, referral

## Abstract

Background

Heart murmurs are defined as whooshing or swishing sounds, unlike the normal lub-dub sounds detected by physicians while using a stethoscope. They usually develop due to numerous pathologies, with congenital defects accounting for the majority of pediatric murmurs. Few studies have addressed the difference in auscultating skills between senior and junior healthcare providers. Therefore, this study aims to collect local data on this topic as well as identify the gap between experienced and inexperienced providers in their ability to accurately detect heart murmurs.

Methodology

This study utilizes a quantitative retrospective design to collect data from King Abdulaziz Medical City and King Faisal Cardiac Centre, Saudi Arabia, from October 1, 2018, to September 30, 2019. The medical records of 292 pediatric patients, who were 14 years of age or below according to the centers’ aging system, were collected from the Hospital Information System (BESTCare). Subsequently, it was determined whether a senior (R3-R4 residents and above) or a junior (R1-R2 residents and interns) healthcare provider ordered an echocardiogram (ECHO). Finally, using the centers’ imaging system (Xcelera) the exact reason for referral, heart murmurs in the case of this study, was obtained, as well as whether a pathologic cause of a murmur was seen in the ECHO image. By obtaining the aforementioned data, the accuracy of each referral was analyzed using statistical analysis software.

Results

ECHO results were categorized into positive and negative outcomes depending on the presence of a structural heart defect, patent foramen ovale (PFO) was considered negative as it causes innocent murmurs. The majority of positive results were atrial septal defects, patent ductus arteriosus, and ventricular septal defects. The majority of negative results were either a structurally normal heart or PFO, which a great number of providers ordered an ECHO for. The Pearson score p-value using the chi-square test was 0.432, leading to the conclusion that junior and senior providers had a similar accuracy of referrals during the study period.

Conclusions

Junior healthcare providers display sufficient knowledge of heart murmur auscultation skills similar to senior healthcare providers during the study period. However, because the data only included two local centers with a limited sample and the absence of further local research on this topic, it is necessary to conduct studies of a larger scope on this topic.

## Introduction

Heart murmurs are defined as whooshing or swishing sounds, unlike the normal lub-dub sounds detected by physicians while using a stethoscope [1,2]. Heart murmurs are occasionally harmless or innocent, asymptomatic, and are mainly caused by increased blood flow to the heart during exercise or growth, such as patent foramen ovale (PFO), and can resolve without medical intervention [3,4]. On the other hand, abnormal heart murmurs indicate heart disease and can either be congenital or develop months or years after birth [5,6]. If symptoms such as poor feeding, shortness of breath, bluish skin, and swelling of the lower legs are observed in children with abnormal heart sounds, the physician may diagnose the child with a heart murmur [7]. While acquired pathological heart murmurs can be caused by several disorders, including fever, infection, anemia, or valve diseases, such as regurgitation or stenosis [8], congenital pathological heart murmurs can be caused mainly by structural defects of the heart that are present at birth. The most common subtypes of congenital heart defects are atrial septal defects (ASDs), ventricular septal defects (VSDs), patent ductus arteriosus (PDA), coarctation of the aorta, cardiac shunts, and valve defects [3,9].

Heart murmurs in children, those under 14 years of age as per most hospitals’ systems, are prevalent in Saudi Arabia, with 13.7 per 1,000 murmurs linked with congenital structural defects (42.5%) [10]. A previous study concluded that VSDs are the most common, accounting for 46.3% of the defects [11]. Following VSD, unspecified congenital heart defects were the second most common, accounting for 27.4% of the cases [12]. The remainder include ASD, cyanotic coronary heart disease (CHD), and non-cyanotic CHD, accounting for 25% of the cases. In addition, studies discussing the incidence of severe CHD have found that most cases are significantly associated with Down syndrome, consanguinity, and maternal diabetes [13]. However, no studies have discussed the impact of CHD on children, and the capabilities of physicians in diagnosing such defects in Saudi Arabia have not been established. Therefore, further local research on the difference in outcomes of children’s heart murmur referrals requested by healthcare providers with disparate experiences needs to be conducted [14].

Previous international studies have been conducted on related subjects. In studies conducted in Canada, the United States, and England, the correct assessment of cardiac murmur ranged between 20% and 26% among residents. However, their percentage was not compared with consultants and other senior physicians [15-17]. On the other hand, a study from Aarhus in Denmark found no correlation between the years of experience of physicians and the ability to accurately identify heart murmurs [18,19]. The sample of this study focused on second- and fifth-year medical students, pre-registration house officers, and cardiologists. In contrast, a research paper written by Cambridge University Press in 2014 included 313 pediatric patients and determined that 60% of those referred for echocardiography (ECHO) had a heart murmur regardless of years of experience [20]. Consequently, the research paper determined that most general practitioner referrals to pediatric cardiologists were accurate. In another study in Canada with a sample of 30 general practice pediatricians, the sensitivity score for the ability to differentiate between pathological and innocent murmurs was 82 (24%), with a mean specificity of 72 (24%) [21-23]. Hence, there was no relationship between diagnostic accuracy and pediatricians’ age, education, or practice characteristics [24]. The study also suggested that the diagnostic accuracy of heart murmurs by general practice pediatricians was suboptimal regardless of years of experience. Thus, further training was required to improve accuracy and reduce inaccurate referrals and misdiagnoses [25].

Currently, few articles have addressed differences in skills between senior and junior healthcare providers in Saudi Arabia. Thus, this research aims to collect local data on the auscultatory skills of providers by determining the difference in the cardiac outcomes of ECHO referrals requested by them depending on their years of experience. This study also hopes to identify the gap between experienced and inexperienced providers in accurately detecting pathologic heart murmurs.

## Materials and methods

Study design, area, and settings

This study is a quantitative cohort retrospective study focused on referrals requested by junior and senior providers for ECHO. This retrospective study design would help form an understanding of the capabilities of juniors and seniors in detecting heart murmurs during the study period. Samples were collected from October 1, 2018, to September 30, 2019, from King Faisal Cardiac Centre (KFCC) and King Abdulaziz Medical Centre (KAMC). To access the aforementioned data, ethical approval was obtained from King Abdullah International Medical Research Center (KAIMARC).

Identification of study participants

The study included the entire population of 292 pediatric patients aged 14 years or below, as per the system’s definition of pediatric patients, who were referred to undergo an ECHO by either junior (R1-R2 and interns) or senior (R3-R4 and above) providers between October 1, 2018, and September 30, 2019. The outcomes of their referral were identified by viewing the ECHO image and distinguishing whether it was positive for a structural defect. It must be noted that even though PFO is a well-known structural defect, it was considered a negative result due to its innocent nature. In contrast, this study excluded referrals for external causes other than heart murmurs and patients whose ECHO results were not added to the Hospital Information System during the study period.

Data collection process

The data were collected by using the Hospital Information System (BESTCare) to obtain the electronic medical records and the computerized physician order entry of pediatric patients referred to perform an ECHO. Subsequently, the centers’ imaging system (Xcelera) to view the result as well as the exact reason for the referral of the ECHO. The BESTCare system also provided data on the patient’s demographic characteristics, such as sex, age, weight, and height, as well as the patient’s medical history.

Data analysis

The collected data related to demographic features were divided into numerical and categorical variables. The numerical variables were described using their means and standard deviations, whereas categorical data were described using the frequency percentage of the obtained samples. In addition, the association between the predictor variable, the senior and junior referral, the outcome variable, and the echocardiogram result was identified using the data analysis software JMP manufactured by the Statistical Analysis System Institute to calculate the chi-square test and frequency of the variables. A p-value of <0.05 was considered significant to determine the correlation between the two variables.

## Results

This study’s population comprised 292 referrals and was divided by age into four main categories. The first category was one week or less, which accounted for 183 (63%) babies. The second category was between one week and three months, which accounted for 37 (13%) babies, and the third was between three months and one year, which accounted for 13 (4%) children. The final category was >one year, which accounted for 58 children and represented 20% of the total population. In addition, 147 (49.7%) referrals were for girls and 145 (50.3%) were for boys. The mean height was 62.4 cm with a standard deviation of 26.8 cm, and the mean weight of the sample was 6.4 kg with a standard deviation of 8.5 kg (Table 1).

**Table 1:** Demographic variables of patients. NA: not available

Demographic variables	Frequency	Percentage
Age		
One week or less	183	63%
One week to three months	37	13%
Three months to 12 months	13	4%
Over one year	58	20%
Unknown	1	NA
Total	291	100%
Gender		
Male	145	49.7%
Female	147	50.3%
Height	62.4 ± 26.8 cm	
Weight	6.4 ± 8.5 kg	

**Figure 1:**
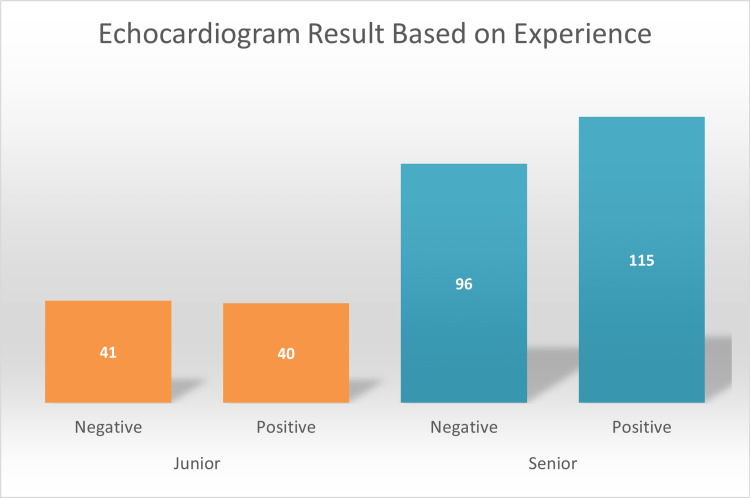
Echocardiogram results based on experience.

Furthermore, ECHO referrals requested by senior providers accounted for 211 (72%) of the cases, while junior providers represented 81 (28%) of the cases (Table 2). Because we included all referrals made during the study period, obtaining a larger junior sample to further equalize the results was impossible. The imaging findings of these referrals identified 383 different results, of which 271 were cardiac abnormalities, and the remaining 112 had structurally normal hearts. The majority of the positive results were ASDs, accounting for 82 (21%) of the cases, followed by PDA (59, 15%), and VSDs (41, 11%). Finally, other cardiac abnormalities were added as a single variable as their percentages were insignificant (25, 7%) (Table 3). On the other hand, the majority of negative results were mainly a structurally normal heart (112, 29%) and PFO (64, 17%) (Table 3). PFOs are structurally different and were counted among the 271 results but were excluded from the overall positive outcomes as they were innocent murmurs. Hence, they were summated with negative ECHO results (Figure 1). To elaborate, of the 64 PFOs, 54 were considered negative as they were not accompanied by other defects.

**Table 2:** Distribution of physicians by years of experience.

Physicians	Frequency	Percentage
Senior	211	72%
Junior	81	28%

**Table 3:** Summary of ECHO results. ECHO: echocardiogram; PFO: patent foramen ovale; VSD: ventricular septal defect; ASD: atrial septal defect; PDA: patent ductus arteriosus

ECHO findings	Frequency	Percentage
Normal	112	29%
PFO	64	17%
VSD	41	11%
ASD	82	21%
PDA	59	15%
Other	25	7%

Ultimately, the study assessed the significance of ECHO results depending on the experience of providers using the chi-square test. Positive ECHO results were similar between junior (40, 49.4%) and senior providers (115, 54.5%). In addition, their false referrals or negative ECHO results were also comparable with juniors, accounting for 41 (50.62%) of the cases, and senior providers accounting for 96 (45.5%) of the cases (Figure 1). Therefore, even though the junior sample was significantly smaller than the senior sample, leading to a larger impact on their accuracy percentage, their positive ECHO result percentage was still fairly similar. Pearson’s p-value using the chi-square test was 0.432. Thus, the accuracy of the auscultatory skills of junior and senior proivders were similar during the study time frame.

## Discussion

Auscultation of heart murmurs is an essential skill that must be acquired by physicians during clinical practice. Therefore, this study aimed to identify the gap in experience between junior and senior providers as local studies on this subject are lacking. After collecting data from KFCC and KAMC using BESTCare and Xcelera, it was determined that there was no association between the providers’ years of experience and the accuracy of their referrals in the study period because the percentage difference in their positive and negative referrals was not significantly varied.

Based on studies conducted in Canada, the United States, and England, the percentage of correct assessments of heart murmurs ranged between 20% and 26% in both junior and senior providers, which is relatively low compared to the findings of this study that revealed a percentage of 49.4% for positive junior providers’ referrals and 54.5% for senior providers [15-17]. The variation in local and previously mentioned international results could be explained by the difference in sample size as international studies included multiple centers and a longer duration of data collection compared to this study. Furthermore, international studies utilized automated methods to collect data, unlike this study, which assembled data manually [24,25].

On the other hand, multiple studies, one of which was conducted in Aarhus, Denmark, and others conducted by Cambridge University, determined that the accuracy of positive referrals for both junior and senior providers was between 50% and 60%, respectively, which is close to the range obtained in this study (49.4-54-5%) [18,19,21,22]. In addition, the difference in accuracy between senior and junior providers in international studies was not significant, mimicking the results of this study. Moreover, these studies had a similar number of PFOs that accounted for a significant number of negative results similar to this study [20]. The aforementioned studies had comparable study designs and data collection methods to this study, which can explain the similarity of the results.

In addition, there are some variations when compared to local studies that determined that VSDs are the most common congenital defects in Saudi Arabia which differ from this study’s outcome which found ASDs to be more common [11,12]. However, overall, it could be deduced that this study, which showed no significant difference in diagnosing heart murmurs based on years of experience, followed the common pattern seen in most international studies. This highlights the reliability of the providers in the previously mentioned centers as the accuracy of both seniors and juniors was similar during the study’s time frame unlike the accuracy of certain international centers [25]. Nonetheless, it must be noted that a few studies report contrasting results to this study [21-23].

The study had a few limitations. First, as the data for this study was collected through BESTCare, which lacked certain information about the providers’ exact year of residency, the potential for a more categorized provider level of experience other than senior and junior was omitted. Second, the reliability of the study is affected by human error as a sample of this size is difficult to manage and review adequately. Third, restricted access to the hospital system might have influenced the ability to extract certain information about the sample’s comorbidities which could have affected the results. Finally, because no similar studies have been conducted in this region, further research on this topic in different centers using other methods and a larger sample size is recommended to acquire more reliable data.

## Conclusions

The percentage of positive and negative results for seniors and juniors was not significantly different. Thus, it can be deduced that juniors displayed sufficient knowledge of heart murmur auscultation skills similar to seniors during the study period. However, because the data only included two local centers with a limited sample and the absence of further local research on this topic, it remains necessary to conduct studies of a larger scope on this topic.
